# Cognitive visual strategies are associated with delivery accuracy in elite wheelchair curling: insights from eye-tracking and machine learning

**DOI:** 10.3389/fpsyg.2025.1682654

**Published:** 2026-01-02

**Authors:** Hongda Zhao, Wei Du, Chao Wang

**Affiliations:** 1 College of Sports Humanities, Harbin Sport University, Harbin, Heilongjiang, China; 2 College of Sports and Art, Harbin Sport University, Harbin, Heilongjiang, China; 3 Graduate School, Harbin Sport University, Harbin, Heilongjiang, China

**Keywords:** wheelchair curling, visual search, para-athletes, eye-tracking, machine learning, delivery accuracy, sport psychology

## Abstract

Visual search is pivotal for athletic performance, yet its role in adaptive sports like wheelchair curling remains understudied. This study investigated how eye-movement features predict delivery accuracy and distinguish elite from novice athletes. Thirty wheelchair curling athletes (15 experts, 15 novices) performed standardized delivery accuracy and visual search tasks, with eye movements recorded using the EyeLink Portable Duo system. We employed multiple regression to identify predictors of accuracy and a support vector machine (SVM) to classify athletes based on expertise. Experts demonstrated superior delivery accuracy and significantly more efficient visual search patterns, characterized by shorter dwell times, faster reaction times, and fewer fixations. The SVM model successfully classified athletes with 90% accuracy (AUC = 0.93), while regression analysis confirmed that specific gaze metrics were robust factors associated with performance. These findings establish a strong quantitative link between efficient gaze strategies and expert motor performance in a constrained-mobility setting. This integrated eye-tracking and machine learning approach offers a powerful framework for objectively evaluating performance and developing data-driven, personalized training interventions in wheelchair curling and other precision-focused adaptive sports.

## 1 Introduction

In dynamic sports, the seamless interplay between visual information processing and motor execution, termed visual-motor coupling, is a cornerstone of athletic performance (Mann et al., 2007; Williams et al., 1999). Grounded in Gibson’s ecological psychology, this process enables athletes to perceive environmental cues and continuously adjust motor responses to meet task demands (Warren, 1990). While extensive research has demonstrated its importance in sports like football and ice hockey (Lochhead et al., 2024; Martell and Vickers, 2004), its role within adaptive sports remains comparatively underexplored. This gap is particularly evident in wheelchair curling, a sport where high precision is paramount and physical mobility is fundamentally constrained, suggesting that visual strategies may play an even more critical role in optimizing performance (Li et al., 2024; Richlan et al., 2023).

Previous research in wheelchair curling has predominantly focused on biomechanical factors, such as release angles and ice friction (Wang et al., 2022). Often overlooking the cognitive-visual contributions to action control. Wheelchair curling presents a unique set of challenges that distinguish it from other sports: athletes must make complex spatial judgments from a fixed, seated position, placing greater neural load on visual-motor integration. This reliance on vision necessitates a deeper understanding of how experts utilize visual search to guide precision delivery (Zacharias et al., 2024). Therefore, building on visual-motor coupling theory, this study aims to elucidate the relationship between specific visual search features and delivery accuracy in wheelchair curling (Koedijker et al., 2010; Holland et al., 2010).

To capture the multifaceted nature of expertise, this study integrates on-field performance assessment with laboratory-based eye-tracking experiments (Hossner and Kredel, 2017). Recognizing the limitations of traditional linear models, we employ a dual-analytic approach: using multiple regression to identify key eye-movement predictors of delivery accuracy, and a support vector machine (SVM) to classify athletes by expertise level based on their gaze behavior (Lou et al., 2017; Shuldiner et al., 2021). We hypothesize that experts, compared to novices, will exhibit more efficient visual search patterns—characterized by shorter dwell times, faster reaction times, and fewer fixations (Panchuk and Vickers, 2006)—and that these patterns will be strongly associated with superior delivery accuracy. Accordingly, this study addresses three core questions: (1) Which specific eye-movement features predict delivery accuracy? (2) How do gaze behaviors and performance differ between expert and novice athletes? (3) How effectively can an SVM model classify expertise based on these visual features?

The primary contribution of this research is twofold. First, while the integration of eye-tracking and machine learning is an emerging trend in sports science, its application has been largely confined to able-bodied sports (Brumann et al., 2021). Its application to a high-precision adaptive sport like wheelchair curling, where visual expertise may play a crucial compensatory role for physical constraints, represents a novel and critical step (Klaib et al., 2021). This study, to our knowledge, is among the first to use a machine learning approach to classify expertise based on cognitive-visual markers in a para-athlete population. Second, by moving beyond simple descriptions of gaze differences, we leverage this integrated approach to identify the key objective markers of expertise.

## 2 Materials and methods

### 2.1 Participants

Thirty wheelchair curling athletes were recruited, comprising 15 experts and 15 novices. The expert group (mean age: 36.8 ± 6.4 years; mean training duration: 10.3 ± 4.3 years) consisted of international-level para-athletes from the Chinese national team, all qualified for the Paralympic Winter Games or World Championships (Burris et al., 2019; Moran et al., 2018). The novice group (mean age: 27.5 ± 4.5 years; mean training duration: 1.2 ± 0.2 years) consisted of amateurs with no formal competition experience. Inclusion criteria for experts were: (1) ≥ 2 years of experience in national-level or higher competitions; (2) ≥ 3 years of systematic training; and (3) classification per International Paralympic Committee standards. For novices, criteria were: (1) no formal competition experience; (2) ≤ 1 year of systematic training; and (3) same classification standards.

An a priori power analysis using G*Power 3.1 for a repeated-measures ANOVA (effect size *f* = 0.25, α=0.05, power = 0.80) indicated a required sample of 28 participants, a threshold met by our sample. All participants had normal or corrected-to-normal vision and provided written informed consent. The study was approved by the Harbin Sports University Ethics Committee (Approval No. 2025013) and adhered to relevant guidelines.

### 2.2 Study design and tasks

#### 2.2.1 Delivery accuracy test

Participants performed eight core delivery techniques (“Side Guard,” “Middle Guard,” etc.) in a standardized curling rink under World Curling Federation guidelines. For each technique, interference stones were placed to simulate competitive scenarios (see Supplementary material 1 for schematics). The final resting position of each delivered stone was recorded by high-definition cameras and scored by evaluators using a standardized 5-point rubric derived from national team standards (see Table 1 for an example). This procedure yielded a performance score for each delivery (Kameda et al., 2020).

**TABLE 1:** Technical evaluation standards for wheelchair curling.

5 Points	4 Points	3 Points	2 Points	1 Point
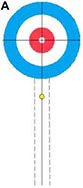 Stone is bisected by the center line.	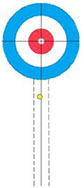 Stone touches the center line.	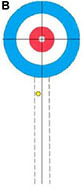 Stone is within the wheelchair lines.	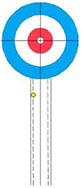 Stone is within the 2-foot lines.	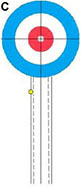 Stone is in Zone 1 or touches the 2-foot line.

This table illustrates the objective and the 5-point scoring rubric for the Middle Guard technique. (A) The primary objective is to place a stone in the Free Guard Zone (Zones 2–3) so that it comes to rest on or near the center line. (B) The scoring rubric visually defines the criteria for scores from 5 points (optimal) to 1 point (minimal success). (C) Scoring Rubric: Schematic Representations and Criteria. All other outcomes are scored as 0 points.

#### 2.2.2 Visual search task

In a controlled laboratory setting, participants viewed 48 static images (Purvis et al., 2015) of different wheelchair curling scenarios. The selection criteria for these images and representative examples are provided in Supplementary material 2, where the images were selected for tactical relevance by five expert coaches. After a 3-s central fixation, each image was presented for 5 s. Participants were instructed to decide on the optimal tactic, pressing “F” for a defensive strategy or “J” for an offensive strategy. Their reaction times and tactical decisions were recorded. This task was designed to isolate the cognitive-perceptual components of decision-making, independent of on-ice motor execution (Roca et al., 2018).

### 2.3 Apparatus

Eye movements for the Visual Search Task were recorded using an EyeLink Portable Duo system (SR Research) at a 1,000 Hz sampling rate (Bachurina and Arsalidou, 2022). The system has a spatial resolution of 0.01 and a precision of 0.15–0.5. Participants’ heads were stabilized with a chin rest to ensure a stable viewing distance of 60 cm. Visual stimuli were presented on a 27-inch HP monitor with a 1,920 × 1,080 pixel resolution and a 120 Hz refresh rate.

### 2.4 Data collection and processing

Prior to the Visual Search Task, a standard nine-point calibration was performed for each participant. Raw eye-movement data were processed using SR Research’s Data Viewer software. Fixations were defined as periods where the eye remained within a 0.5° visual angle dispersion for at least 100 ms. Saccades were detected using a velocity threshold of 30°/s and an acceleration threshold of 8,000°/s^2^, parameters commonly used to ensure reliable event detection in EyeLink systems (Ehinger et al., 2019). Data quality was confirmed by analyzing the saccadic main sequence, which revealed a strong positive correlation between saccade amplitude and peak velocity for both expert (*r* = 0.91) and novice (*r* = 0.88) groups, consistent with established standards for high-quality eye-tracking data (Bahill et al., 1975).

### 2.5 Data analysis

All statistical analyses were conducted using SPSS 25.0.

#### 2.5.1 Performance and behavioral analysis

Independent samples *t*-tests were used to compare delivery accuracy scores, reaction times, and tactical decision accuracy between expert and novice groups (Field, 2017). Effect sizes were calculated using Cohen’s *d*. A 2 (Group: expert vs. novice) × 2 (Tactical Condition: offensive vs. defensive) repeated-measures ANOVA was conducted on eye-movement metrics (e.g., fixation count, dwell time). The Bonferroni correction was applied for all *post-hoc* comparisons.

#### 2.5.2 Predictive modeling

A dual-analytic approach was used to model the relationship between gaze behavior and expertise.

Multiple Regression: A stepwise multiple regression analysis was performed to identify which eye-movement and behavioral variables were significant predictors of delivery accuracy.

Support Vector Machine (SVM) Modeling: An SVM model with a Radial Basis Function (RBF) kernel was developed in Python (scikit-learn) to classify athletes as “expert” or “novice.” The model was trained on a comprehensive set of 10 predictive features derived from both the on-ice and laboratory tasks. These features encompassed three domains: (a) performance metrics (overall delivery accuracy and tactical decision accuracy); (b) a behavioral metric (reaction time); and (c) a suite of seven core eye-movement metrics, including mean fixation duration, saccade count, average saccadic amplitude, and key area-of-interest (AOI) specific parameters such as fixation count and time to first fixation. All features were standardized using Min-Max scaling. A 10-fold cross-validation with a grid search was used to optimize hyperparameters (C and gamma), with the Area Under the Curve (AUC) serving as the primary metric for model selection (Gong et al., 2025; Chen et al., 2020).

## 3 Results

### 3.1 Delivery accuracy: group differences

**FIGURE 1:**
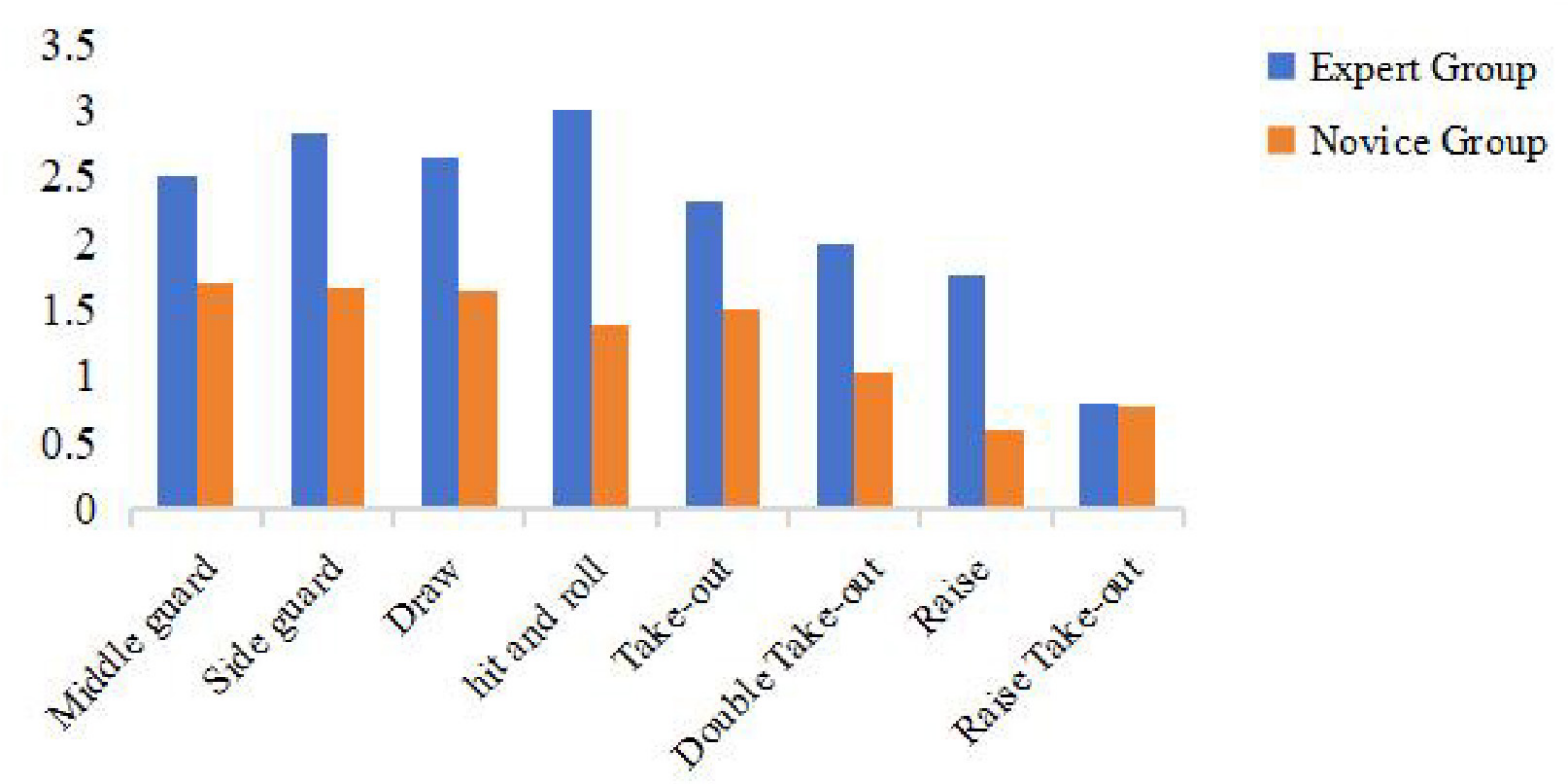
Mean delivery accuracy scores across eight techniques for expert and novice athletes. Description: Bar graph comparing mean delivery accuracy scores for eight techniques between expert and novice groups.

The primary analysis of on-ice performance revealed a significant expertise-related difference in delivery accuracy. As detailed in Table 2, the expert group (*M* = 45.01, SD = 7.15) achieved a significantly higher overall mean score than the novice group (*M* = 26.23, SD = 7.84; *t*(28) = 6.858, *p* < 0.001). Figure 1 provides a visual representation of this performance gap, illustrating that the experts’ superiority was a consistent pattern across the majority of the tasks. This finding is further supported by the analysis of performance stability. The coefficient of variation (CV), as shown in Table 3, was markedly lower for the expert group (15.91%) compared to the novice group (29.91%), indicating that experts performed not only with higher accuracy but also with significantly greater consistency, a hallmark of elite performance developed through extensive practice (Ericsson et al., 1993).

**TABLE 2:** Delivery accuracy scores and *t*-test results.

Index	Expert group	Novice group	*T*	*P*
Delivery accuracy	45.01 ± 7.15	26.23 ± 7.84	6.858	<0.001

**TABLE 3:** Coefficient of variation (CV) for delivery accuracy.

Index	Expert group	Novice group
Coefficient of variation (%)	15.91	29.91

### 3.2 Technique-specific analysis of delivery accuracy

To further explore the nature of the experts’ superiority, we analyzed performance differences for each of the eight delivery Techniques (see Figure 1). The results indicate that the performance gap between experts and novices varied depending on the technical and tactical complexity of the shot. The largest and most significant differences were observed in high-precision, complex techniques. For instance, in the “Hit and Roll” task, experts (*M* = 2.50) scored more than double that of novices [*M* = 1.20; *t*(28) = 5.12, *p* < 0.001, Cohen’s *d* = 1.92]. Similarly large advantages for the expert group were found in the “Side Guard” [*t*(28) = 4.25, *p* < 0.001, Cohen’s *d* = 1.72] and “Middle Guard” [*t*(28) = 3.98, *p* < 0.01, Cohen’s *d* = 1.50] techniques, which require precise control of both weight and line.

In contrast, the performance gap narrowed for techniques perceived as less complex. While still significant, the difference was smaller for the “Raise” technique [*t*(28) = 2.34, *p* = 0.026, Cohen’s *d* = 0.88]. Notably, for the “Raise Take-Out” technique, the difference was not statistically significant [*t*(28) = 1.87, *p* = 0.071, Cohen’s *d* = 0.70], with both groups performing similarly, suggesting a universally high level of difficulty for this specific task.

### 3.3 Tactical decision accuracy and reaction time in the visual search task

**FIGURE 2:**
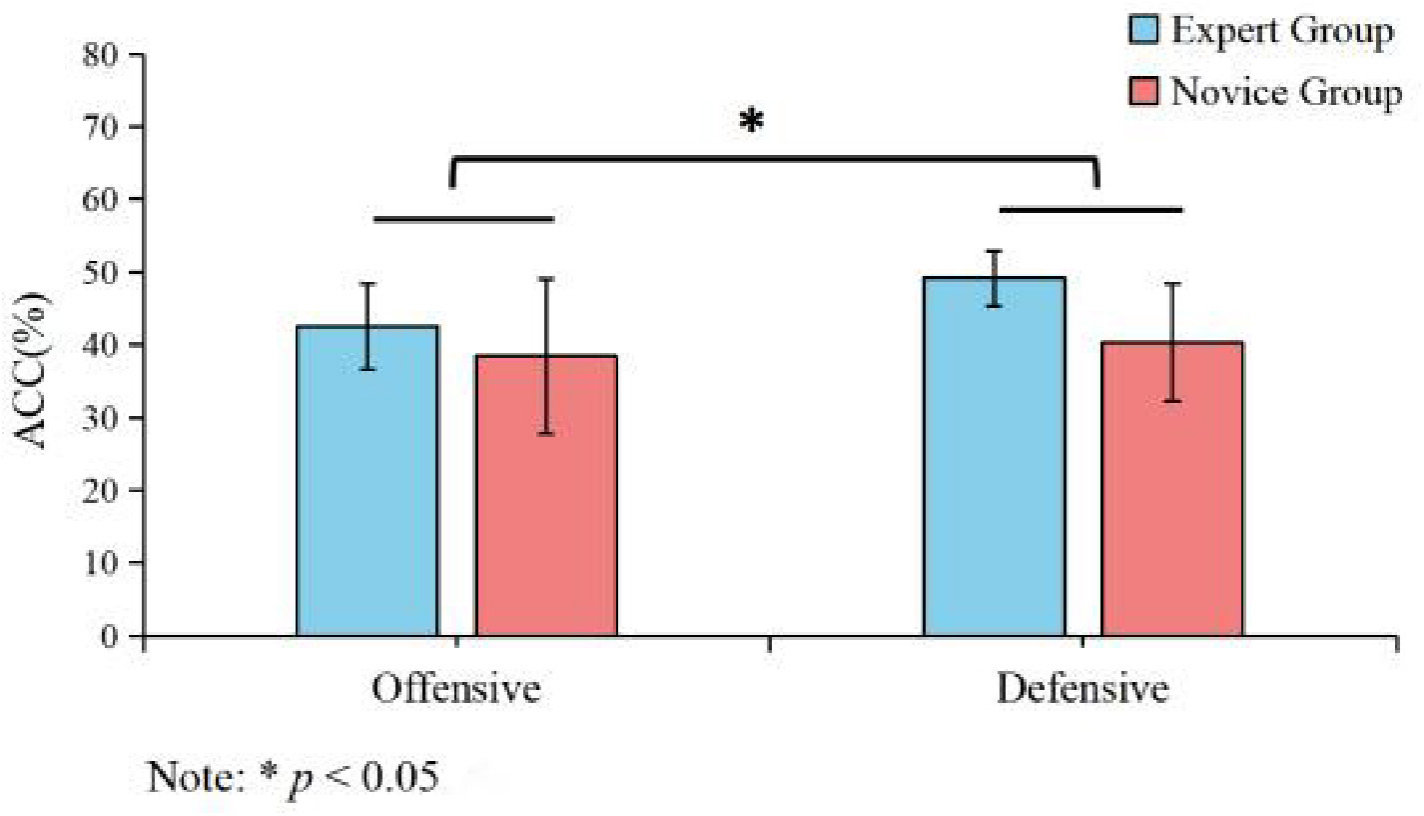
Tactical decision accuracy rates for expert and novice athletes under offensive and defensive conditions. Description: Line graph showing accuracy rates under different tactical conditions. *p < 0.05.

A 2 (Group: expert vs. novice) × 2 (Tactical Condition: offensive vs. defensive) repeated-measures ANOVA was performed on the tactical accuracy data. While the analysis revealed no significant main effect of Group [*F*(1, 28) = 1.432, *p* = 0.241], it did show a significant main effect of Tactical Condition [*F*(1, 28) = 4.643, *p* = 0.04, η*_*p*_*^2^ = 0.142]. Crucially, these effects were superseded by a strong and significant Group × Tactical Condition interaction [*F*(1, 28) = 11.391, *p* = 0.002, η*_*p*_*^2^ = 0.289], as depicted in Figure 2. *Post-hoc* analysis to decompose this interaction confirmed that the experts’ advantage was specific to the more cognitively demanding defensive scenarios. Here, experts (*M* = 49.15%, SD = 3.81) were significantly more accurate than novices (*M* = 40.30%, SD = 8.09; *p* < 0.05). This indicates that the tactical superiority of experts is not a general trait but one that emerges specifically under high cognitive load (Roca et al., 2018).

**FIGURE 3:**
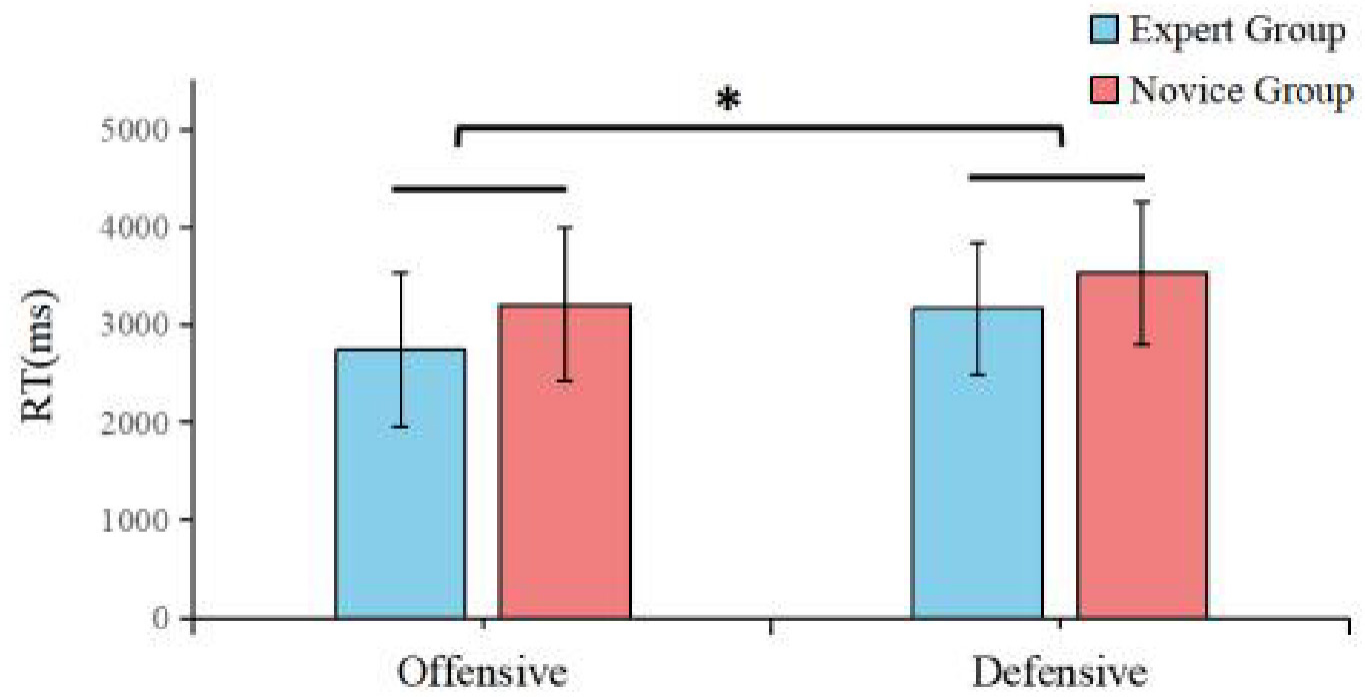
Reaction times for expert and novice athletes under offensive and defensive conditions. *p < 0.05.

Analysis of reaction time (RT) revealed a significant main effect of Tactical Condition, with both groups responding more slowly in defensive tasks [*F*(1, 28) = 4.81, *p* =.037, η*_*p*_*^2^ = 0.147]. As illustrated in Figure 3, there was a marginally significant main effect of Group, suggesting a trend for experts to be faster than novices overall [*F*(1, 28) = 3.754, *p* = 0.063, η*_*p*_*^2^ = 0.118]. *Post-hoc* data confirmed that experts consistently responded faster than novices across both offensive (Expert *M* = 2742.81 ms; Novice *M* = 3205.92 ms) and defensive conditions (Expert *M* = 3169.02 ms; Novice *M* = 3535.47 ms), demonstrating a consistent advantage in decision-making efficiency.

### 3.4 Gaze behavior analysis: markers of enhanced visual efficiency

To investigate the underlying mechanisms of expert performance, a detailed analysis of participants’ gaze behaviors was conducted. The results revealed a consistent pattern of enhanced visual efficiency in the expert group.

#### 3.4.1 Superior search efficiency

**FIGURE 4:**
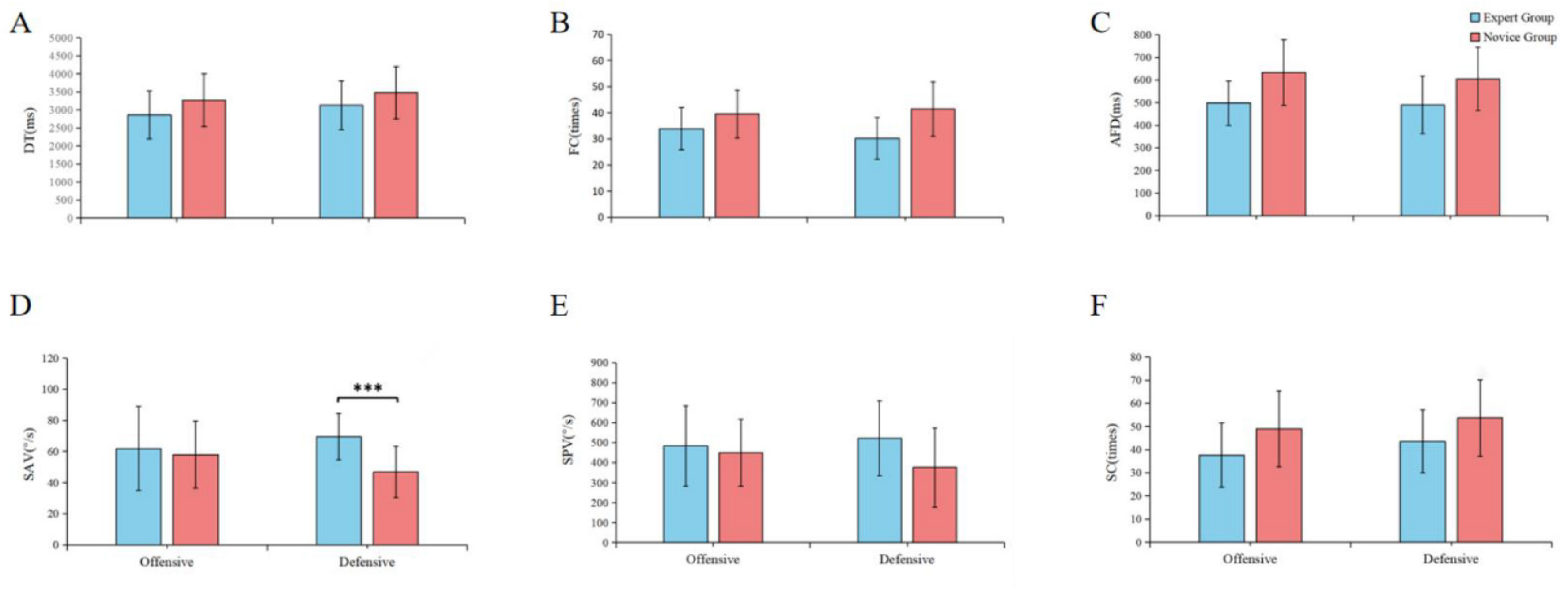
Group differences in key visual search metrics. Description: Comparison of visual search metrics between expert (blue bars) and novice (red bars) athletes under offensive and defensive conditions. Error bars represent standard deviation. ***p < 0.001. **(A)** Total dwell time (DT). **(B)** Fixation count (FC). **(C)** Average fixation duration (AFD). **(D)** Saccadic average velocity (SAV). **(E)** Saccadic peak velocity (SPV). **(F)** Saccadic count (SC).

Experts employed a more efficient visual search strategy, which is a primary indicator of visual expertise. They exhibited a significantly lower total fixation count than novices across both tactical conditions [main effect of Group: *F*(1, 28) = 15.747, *p* < 0.001, η*p*^2^ = 0.360; Figure 4A]. No significant statistical differences were observed in the data presented in Figure 4B. Correspondingly, their average fixation duration was also significantly shorter [main effect of Group: *F*(1, 28) = 13.898, *p* = 0.001, η*_*p*_*^2^ = 0.332; Figure 4C]. Taken together, these findings indicate that experts are more adept at quickly identifying and processing key visual cues, requiring fewer and briefer “snapshots” of the visual scene (Martell and Vickers, 2004; Panchuk and Vickers, 2006).

#### 3.4.2 Faster information processing and saccadic control.

Beyond fixation patterns, experts also demonstrated faster overall information processing. This was reflected in the total dwell time, where a trend toward shorter durations was observed for the expert group [main effect of Group: *F*(1, 28) = 3.343, *p* = 0.078, η*_*p*_*^2^ = 0.107; Figure 4D]. This rapid processing was supported by more effective saccadic control. Experts exhibited significantly faster saccadic average velocity [*F*(1, 28) = 5.559, *p* < 0.05, η*_*p*_*^2^ = 0.166; Figure 4E] and made significantly fewer saccades overall [saccadic count: *F*(1, 28) = 6.428, *p* < 0.05, η*_ρ_* ^2^ = 0.187]. This suite of metrics suggests that experts not only process information more quickly at each fixation point but also move their eyes more rapidly and purposefully between points of interest (Jin et al., 2023).

#### 3.4.3 A Nuanced strategy: the counterintuitive role of the first fixation

**FIGURE 5:**
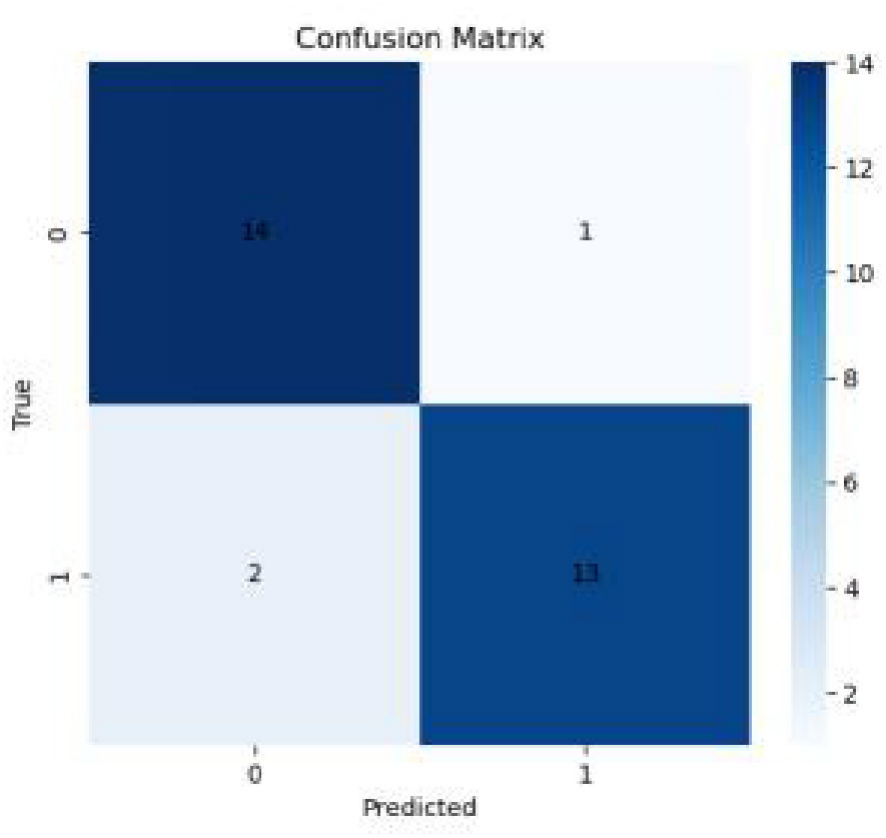
Confusion matrix of the SVM model for visual search features.

Interestingly, this pattern of global efficiency was nuanced by a more complex initial analysis strategy. The analysis of first fixation duration (FFD) revealed a significant Group × Tactical Condition interaction [*F*(1, 28) = 4.674, *p* = 0.039, η*_*p*_*^2^ = 0.143]. As shown in Figure 5, in the more cognitively demanding defensive scenarios, experts’ first fixations were significantly longer than those of novices. This counterintuitive finding suggests a sophisticated expert strategy: a strategic “cognitive pause” for deeper initial analysis of the most critical information before proceeding with an otherwise rapid and efficient search (Klostermann and Hossner, 2018).

### 3.5 Predictive modeling of performance and expertise

#### 3.5.1 Multiple regression: predicting delivery accuracy

To identify which cognitive-visual features were most strongly associated with on-ice delivery accuracy, a stepwise multiple regression analysis was conducted. To ensure the robustness of the model given the sample size, we employed a non-parametric bootstrapping procedure (1,000 resamples) to assess the stability of the regression coefficients. The analysis revealed that second fixation entry time (β = −0.45, *p* < 0.01) and saccadic speed (β = 0.32, *p* < 0.05) were significant and stable predictors. This indicates that faster initial fixations and quicker saccades are robustly associated with higher accuracy in experts.

#### 3.5.2 SVM: classifying expertise level

**FIGURE 6:**
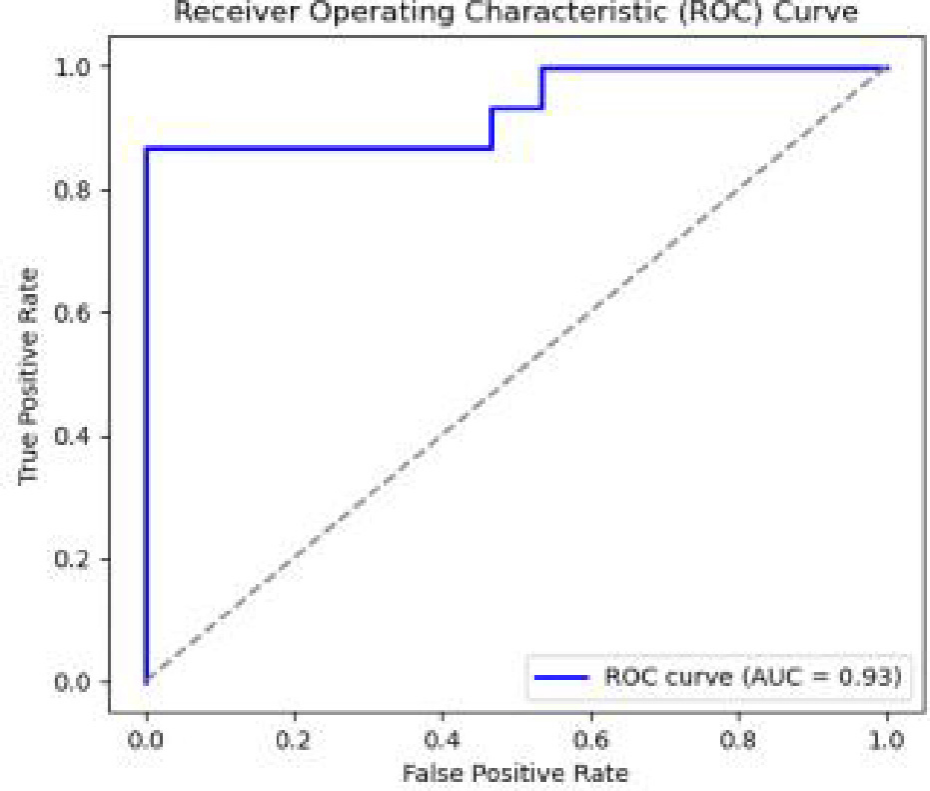
The ROC curve of the SVM model for visual search features.

An SVM model was developed to classify athletes based on their cognitive-visual features. Acknowledging that leave-one-out (LOO) cross-validation can yield optimistic estimates, we performed a more rigorous validation using a bootstrapped 10-fold cross-validation procedure (Jiang et al., 2013). This robust validation yielded a mean classification accuracy of 88.5% with a 95% confidence interval of (82.1–94.9%). The model achieved a mean Area Under the Curve (AUC) of 0.882, indicating excellent discriminative power. This approach of using machine learning to classify skill levels based on eye-movement data is gaining traction and has proven effective in other domains (Lou et al., 2017; Shuldiner et al., 2021). This performance was confirmed to be significantly greater than chance by a permutation test (*p* < 0.001). The model’s performance is further visualized in the confusion matrix (Figure 6) and the ROC curve (Figure 6).

## 4 Discussion

This study provides novel insights into the cognitive-visual strategies that distinguish expert and novice wheelchair curling athletes. Our findings reveal a clear pattern of expertise: experts not only exhibit superior delivery accuracy but also employ significantly more efficient visual search patterns. However, a deeper analysis reveals that this superiority is nuanced and context-dependent. Experts’ advantages in tactical decision-making and initial visual analysis emerge most strongly under conditions of high cognitive load, such as complex defensive scenarios. The discussion below integrates these empirical findings with established theoretical frameworks to explain the underlying perceptual-cognitive mechanisms (Williams et al., 1999).

### 4.1 Expert gaze behavior as attunement to affordances

Our central finding—that experts process visual information more efficiently—can be powerfully interpreted through the lens of Gibson’s ecological psychology. According to this framework, skilled perception involves becoming “attuned” to the “affordances” for action that an environment provides (Warren, 1990). The curling sheet is rich with affordances: the open path to the button affords a “draw” shot, while the position of an opponent’s stone affords a “take-out.” Our results provide concrete behavioral markers for this process of direct perception. The experts’ significantly faster initial fixations are not merely a sign of quicker eye movements; they represent the immediate identification of the most critical affordance on the sheet without conscious, step-by-step analysis (Gibson, 1979; Fajen et al., 2009). Furthermore, their shorter dwell times suggest that once an affordance is perceived—for example, the precise angle for a “Hit and Roll” behind a guard—they require minimal cognitive effort to confirm its viability. They “see” the possibility and almost instantly move to planning the execution. Novices, in contrast, must engage in a more effortful, feature-by-feature analysis, reflected in their longer and more numerous fixations. Their gaze lingers as they consciously calculate angles, distances, and potential outcomes, indicating a perceptual system not yet finely tuned to the actionable possibilities of the game (Araújo et al., 2006).

### 4.2 Cognitive mechanisms: pattern recognition and chunking

The efficiency of expert gaze can also be explained by well-established cognitive mechanisms of expertise (Hossner and Kredel, 2017). The shorter dwell times observed in our expert group are consistent with superior pattern recognition abilities. Experts are not just seeing individual stones; they are rapidly recognizing familiar patterns of play that they have encountered thousands of times. This allows them to “chunk” complex visual information into larger, meaningful units. This “chunking” process is directly observable in our eye-tracking data. For instance, a complex tactical situation—such as a high center guard protecting two of their own stones biting the top of the eight-foot circle—is not processed by an expert as three separate objects requiring three distinct fixations. Instead, it is immediately perceived as a single, recognizable tactical pattern: a “well-constructed defensive fortress.” This is why experts demonstrated significantly fewer fixations; their gaze pattern efficiently captures the entire strategic gestalt in one or two saccades. This chunking process drastically reduces cognitive load, freeing up mental resources for higher-level strategic planning (Ericsson and Kintsch, 1995). This cognitive advantage is directly reflected in our predictive models (Chase and Simon, 1973; Reingold et al., 2001). The multiple regression analysis revealed that tactical decision accuracy and reaction time were among the strongest predictors of on-ice delivery performance. This provides a quantitative link: the ability to rapidly chunk visual patterns is what enables the faster, more accurate decisions that ultimately is related to a successful shot.

### 4.3 The integrated perceptual-cognitive system: a necessary adaptation to the constraints of wheelchair curling

Neither the direct perception of affordances (section 4.1) nor the cognitive mechanism of chunking (section 4.2) operates in isolation. Instead, they form a synergistic and highly integrated perceptual-cognitive system. We argue that chunking serves as the perceptual foundation upon which the direct perception of higher-order affordances is built. It is only after a complex arrangement of stones is chunked from individual, meaningless objects into a recognizable tactical pattern (e.g., a “center-line block”) that an expert can then directly perceive the sophisticated affordances for action, such as the narrow possibility for a “thread-the-needle” shot. The novice, unable to perform the initial chunking, remains perceptually blind to these advanced tactical opportunities.

Crucially, the significance of this integrated system is magnified by the unique physical constraints inherent to wheelchair curling. Unlike their able-bodied counterparts, who can physically ambulate around the house, change their viewing angle, and use parallax to build a rich, three-dimensional understanding of the stone arrangement, wheelchair athletes are largely restricted to a single, static, and low-level viewpoint. This physical limitation places an enormous demand on their cognitive-visual system (Flueck, 2020). All the complex spatial judgments—calculating angles, visualizing stone trajectories, and mentally rotating the entire scene to anticipate post-shot outcomes—must be performed internally, without the aid of physical repositioning (Voyer et al., 1995).

Therefore, the highly efficient visual search patterns we observed in experts are not merely a hallmark of general sporting expertise; they are a highly specialized and necessary adaptation. Their ability to extract maximum strategic information with minimal eye movements serves as a powerful compensatory mechanism for their physical immobility (Salthouse, 1984). Unable to change their viewpoint physically, they have developed a “mental rotation” and “perceptual chunking” system that is so advanced it allows them to build a robust and accurate mental model of the game from a single, static vantage point (Ericsson and Lehmann, 1996). This underscores the critical importance of visual expertise as a core compensatory skill (Causer et al., 2017).

### 4.4 Limitations

Several limitations inherent to the study’s design warrant consideration. The cross-sectional nature of the research establishes strong associations but precludes causal inferences regarding the development of expertise (Shadish et al., 2002). Furthermore, the reliance on static 2D images, while ensuring experimental control, does not fully replicate the dynamic perceptual-motor environment of on-ice gameplay, thus constraining the study’s ecological validity (Dicks et al., 2010). The generalizability of the findings is also limited by the specific context of wheelchair curling and a sample size that, while robust for this specialized population (Ericsson and Lehmann, 1996), remains small in absolute terms. This is compounded by the use of a distinct expert-novice dichotomy, which, though effective for maximizing group contrast, does not capture the full continuum of skill development (Feltovich et al., 2006).

## 5 Conclusion

This study provides a comprehensive quantitative analysis of the cognitive-visual strategies underlying expertise in wheelchair curling, establishing a strong link between efficient visual search and superior delivery accuracy through the integration of on-ice performance metrics, eye-tracking, and machine learning. Our findings demonstrate that expert wheelchair curlers exhibit a distinct and more efficient gaze profile, characterized by fewer fixations, shorter overall fixation durations, and faster saccadic eye movements. Critically, we also identified a more nuanced aspect of their strategy: a longer initial fixation in complex defensive scenarios, suggesting a deeper initial analysis of the tactical landscape. The power of these cognitive-visual features as objective markers of expertise was confirmed by our SVM model, which distinguished experts from novices with 90% accuracy (AUC = 0.93).

In summary, this research highlights that expertise in wheelchair curling is not solely a matter of refined motor execution but is critically underpinned by a highly adapted perceptual-cognitive system. The data-driven approach presented here offers a valuable framework for performance assessment and provides a methodological blueprint for moving beyond traditional observational coaching, particularly within the field of adaptive sports. By demonstrating how quantitative eye-tracking data can be leveraged by machine learning, our work paves a new path for developing evidence-based, technology-driven training interventions tailored to the unique needs of para-athletes.

While this study establishes a robust foundation, it also illuminates the path for future investigations essential for advancing the field. To establish a causal link between gaze behavior and performance, future work should prioritize longitudinal designs to track the natural development of visual strategies. Targeted training interventions, such as gaze-contingent feedback and virtual reality (VR) simulations, could then be employed to directly evaluate and enhance skill acquisition. Furthermore, enhancing ecological validity through in-game mobile eye-tracking and systematically testing the generalizability of these findings through cross-sport comparative studies are crucial next steps. Finally, expanding the machine learning approach to integrate other data streams, such as pupillometry, will allow for a more comprehensive and predictive understanding of the multifaceted nature of expertise in adaptive sports. Pursuing these directions will not only deepen our theoretical knowledge but also transform how we analyze performance and cultivate talent in this important domain.

## Data Availability

The raw data supporting the conclusions of this article will be made available by the author, without undue reservation.
