# Supplementary material for: Cognitive visual strategies are associated with delivery accuracy in elite wheelchair curling: insights from eye-tracking and machine learning
Source: Front Psychol. 2026 Jan 2;16:1682654. doi: 10.3389/fpsyg.2025.1682654 (PMC12808461; doi:10.3389/fpsyg.2025.1682654)
Supplement: Supplementary file 1 [file Data_Sheet_1.pdf]

Supplementary Material 1: Detailed Scoring Protocols for the Eight Core Wheelchair Curling Techniques

Figure S1. Scoring Protocol for the "Middle Guard" Technique.

This figure illustrates the objective and the 5-point scoring rubric for the Middle Guard technique. (A) The primary objective is to place a stone in the Free Guard Zone (Zones 2–3) so that it comes to rest on or near the center line. (B) The scoring rubric visually defines the criteria for scores from 5 points (optimal) to 1 point (minimal success).

(B) Scoring Rubric: Schematic Representations and Criteria

5 Points	4 Points	3 Points	2 Points	1 Point
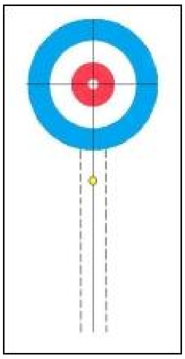	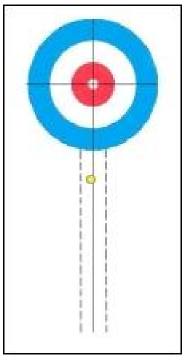	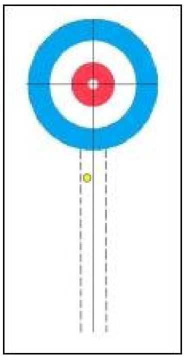	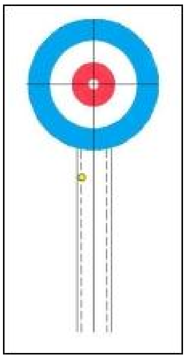	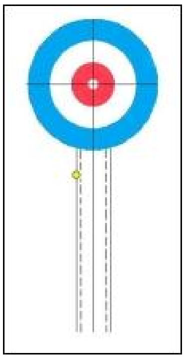
Stone is bisected by the center line.	Stone touches the center line.	Stone is within the wheelchair lines.	Stone is within the 2-foot lines.	Stone is in Zone 1 or touches the 2-foot line.

Note: *All other outcomes are scored as 0 points.*

Figure S2. Scoring Protocol for the "Side guard" Technique.

This figure illustrates the objective and the 5-point scoring rubric for the Side Guard technique. (A) The primary objective is to place a stone in the Free Guard Zone (Zones 2–3) so that it comes to rest to the side of the center line, acting as a guard. (B) The scoring rubric visually defines the criteria for scores from 5 points (optimal) to 1 point (minimal success).

(B) Scoring Rubric: Schematic Representations and Criteria

5 Points	4 Points	3 Points	2 Points	1 Point
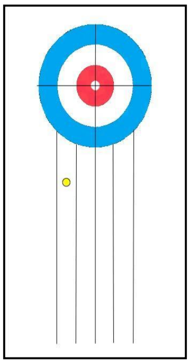	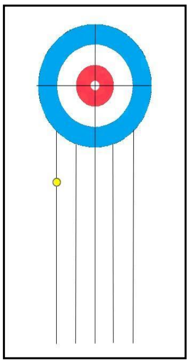	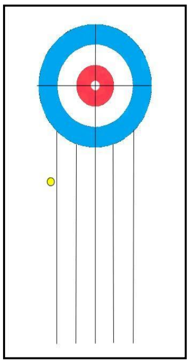	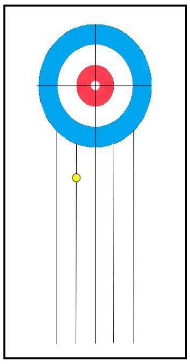	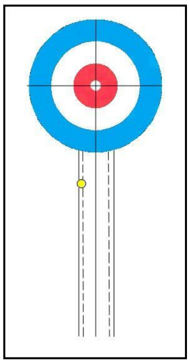
Between 2 and 4 feet from the center line	Stone touches the 4-foot line.	Between 4 and 6 feet from the center line.	Stone touches the 2-foot line.	Stone touches the wheelchair line.

Note: *All other outcomes are scored as 0 points.*

Figure S3. Scoring Protocol for the "Draw" Technique.

This figure illustrates the objective and the 5-point scoring rubric for the Draw technique. (A) The primary objective is to deliver a stone with the correct weight so that it comes to rest within the scoring area (the house). (B) The scoring rubric visually defines the criteria for scores from 5 points (optimal) to 1 point (minimal success).

(B) Scoring Rubric: Schematic Representations and Criteria

5 Points	4 Points	3 Points	2 Points	1 Point
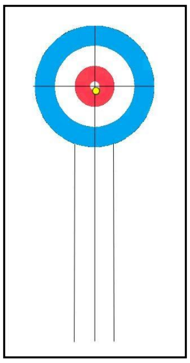	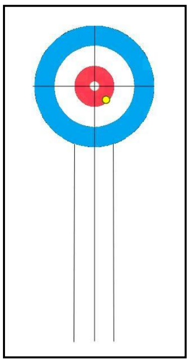	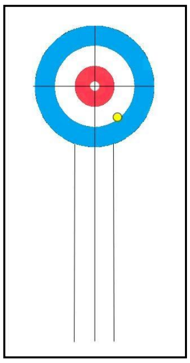	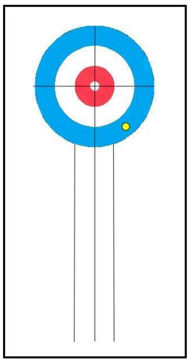	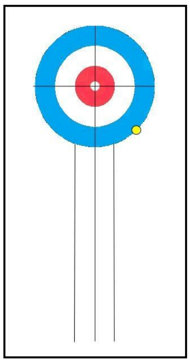
Stone touches the 6-inch ring (button).	Stone touches the 2-foot circle.	Stone touches the 4-foot circle.	Stone is between the 4 and 6-foot circles.	Stone touches the 6-foot circle.

Note: *All other outcomes are scored as 0 points.*

Figure S4. Scoring Protocol for the "Hit and roll" Technique.

This figure illustrates the objective and the 5-point scoring rubric for the Hit and Roll technique. (A) The primary objective is to hit a designated target stone, removing it from play, and have the shooter's stone roll to a specific scoring position within the house. (B) The scoring rubric visually defines the criteria for scores from 5 points (optimal) to 1 point (minimal success).

(B) Scoring Rubric: Schematic Representations and Criteria

5 Points	4 Points	3 Points	2 Points	1 Point
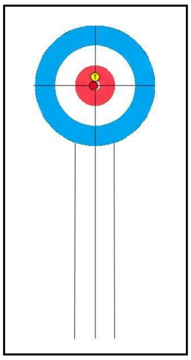	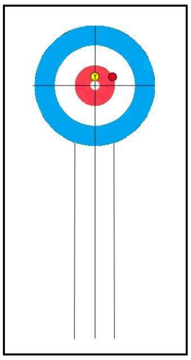	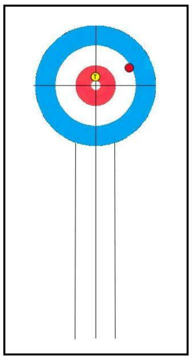	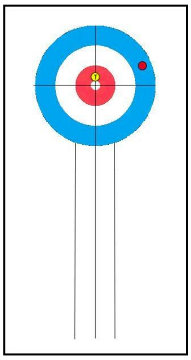	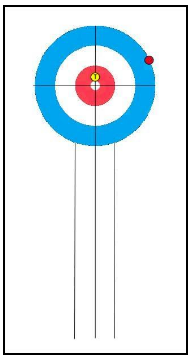
Shooter stone touches the 6-inch ring after contact.	Shooter stone touches the 2-foot circle after contact.	Shooter stone touches the 4-foot circle after contact.	Shooter stone is between the 4-6 foot circles after contact.	Shooter stone touches the 6-foot circle after contact.

Note: *All other outcomes (e.g., missing the target stone) are scored as 0 points.*

Figure S5. Scoring Protocol for the "Take-out" Technique.

This figure illustrates the objective and the 5-point scoring rubric for the Take-out technique. (A) The primary objective is to remove a designated target stone from play, while controlling the position of the shooter stone. The initial setup involves two potential opponent's target stones, which are depicted as red, dashed circles to indicate their pre-shot positions: Target 1 (a guard on the center line) and Target 2 (a stone in the 2-foot circle). (B) The scoring rubric visually defines the criteria based on the final resting position of the shooter stone (our solid yellow stone) after it successfully removes a target.

(B) Scoring Rubric: Schematic Representations and Criteria

5 Points	4 Points	3 Points	2 Points	1 Point
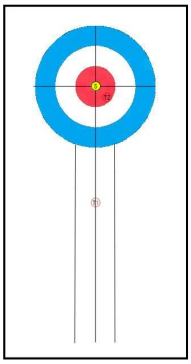	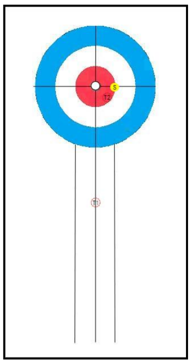	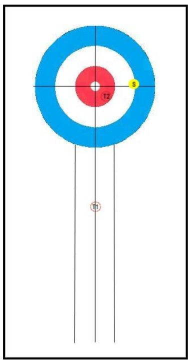	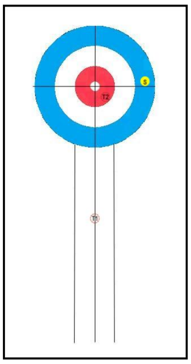	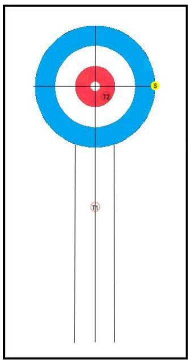
Shooter stone rolls to the 6-inch ring after contact.	Shooter stone rolls to the 2-foot circle after contact.	Shooter stone rolls to the 4-foot circle after contact.	Shooter stone is between the 4-6 foot circles after contact.	Shooter stone rolls to the 6-foot circle or remains in the house.

Note: *All other outcomes (e.g., missing the target stone) are scored as 0 points.*

Figure S6. Scoring Protocol for the "Double Take-out" Technique.

This figure illustrates the objective and the 5-point scoring rubric for the Double Take-out technique. (A) The primary objective is to remove two designated opponent's target stones from play with a single delivery. The initial setup places Target 1 (T1) and Target 2 (T2) at their pre-shot positions, which are depicted as red, dashed circles. (B) The scoring rubric visually defines the criteria based on the outcomes for all three stones. In the schematics, a green checkmark (√) over a target stone indicates it was successfully removed from play. The shooter stone is depicted as a solid yellow stone if it remains in the house, or a hollow yellow stone if it rolls out.

(B) Scoring Rubric: Schematic Representations and Criteria

5 Points	4 Points	3 Points	2 Points	1 Point
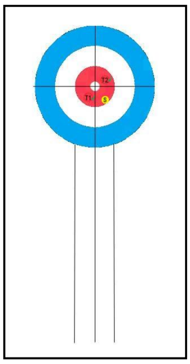	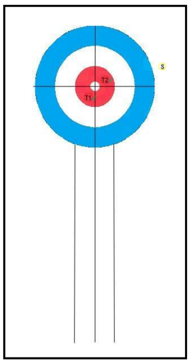	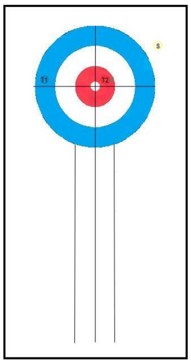	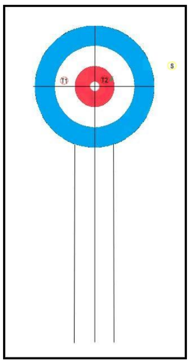	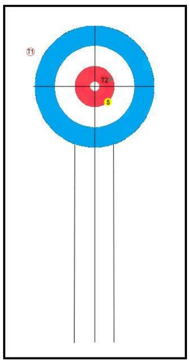
Both target stones removed from play and shooter stone remains in the house.	Both target stones removed from play, but shooter stone rolls out of the house.	One target stone removed; the second is moved but remains outside the 4-foot circle.	One target stone removed; the second is moved but remains inside the 4-foot circle.	One target stone removed and the shooter stone remains in the house.

Note: *All other outcomes are scored as 0 points.*

Figure S7. Scoring Protocol for the "Raise" Technique.

This figure illustrates the objective and the 5-point scoring rubric for the Raise technique. (A) The primary objective is to use the shooter stone (S) to tap one of the team's own pre-positioned stones (Target 1) into the house to score. This is a cooperative play between two of the team's own stones. (B) The scoring rubric visually defines the criteria based on the final resting position of Target 1. In the schematics, the initial position of our target stone is shown as a yellow, dashed circle, while its final, scoring position is shown as a solid yellow stone.

(B) Scoring Rubric: Schematic Representations and Criteria

5 Points	4 Points	3 Points	2 Points	1 Point
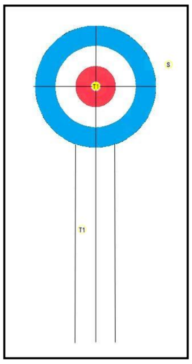	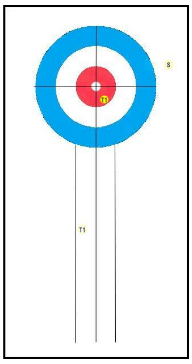	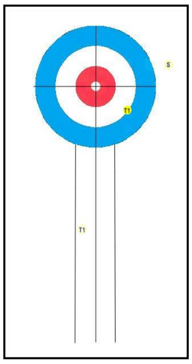	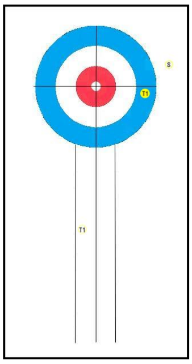	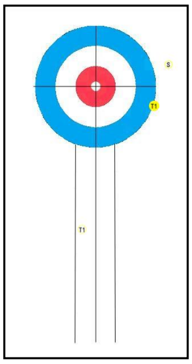
Target stone rolls to the 6-inch ring after contact.	Target stone rolls to the 2-foot circle after contact.	Target stone rolls to the 4-foot circle after contact.	Target stone is between the 4-6 foot circles after contact.	Target stone rolls to the 6-foot circle after contact.

Note: *All other outcomes are scored as 0 points.*

Figure S8. Scoring Protocol for the "Raise Take-out" Technique.

This figure illustrates the objective and the 5-point scoring rubric for the Raise Take-out technique. (A) The primary objective is to use the shooter stone to raise one of the team's own stones (Target 1) into the house to remove an opponent's stone (Target 2). (B) The scoring rubric visually defines the criteria based on the outcome for both target stones. In the schematics, initial positions are shown as dashed circles (yellow for our stone, red for the opponent's). A green checkmark (√) indicates the successful removal of the opponent's stone, while the final position of our stone is depicted by a solid yellow stone.

(B) Scoring Rubric: Schematic Representations and Criteria

5 Points	4 Points	3 Points	2 Points	1 Point
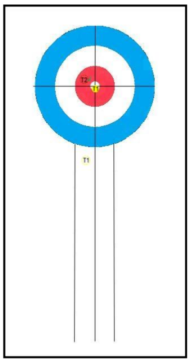	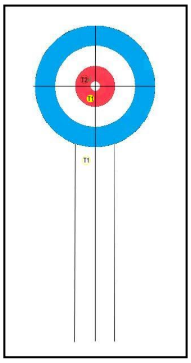	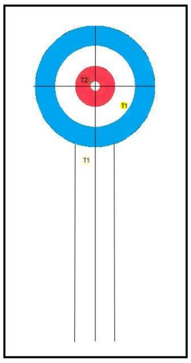	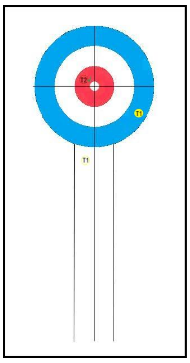	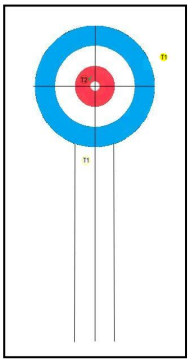
Opponent's stone removed and our stone stops in the 6-inch ring.	Opponent's stone removed and our stone stops in the 2-foot circle.	Opponent's stone removed and our stone stops in the 4-foot circle.	Opponent's stone removed and our stone stops in the 6-foot circle.	Opponent's stone removed, but our stone also rolls out of the house.

Note: *All other outcomes are scored as 0 points.*
