# Supplementary material for: Cognitive visual strategies are associated with delivery accuracy in elite wheelchair curling: insights from eye-tracking and machine learning
Source: Front Psychol. 2026 Jan 2;16:1682654. doi: 10.3389/fpsyg.2025.1682654 (PMC12808461; doi:10.3389/fpsyg.2025.1682654)
Supplement: Supplementary file 2 [file Data_Sheet_2.pdf]

Supplementary Material 2: Representative Examples and Selection Criteria for Visual Stimuli

Image Selection Criteria

The 48 visual stimuli used in this study were selected from a large pool of real-game photographs to ensure high ecological validity. The selection process was conducted by a panel of five experts, including senior coaches and athletes from the national wheelchair curling team. The final set of images was chosen based on the following comprehensive criteria:

Tactical Diversity: The images covered a wide and balanced range of common tactical scenarios, including but not limited to: Offensive situations (e.g., setting up for a multi-point end), Defensive situations (e.g., placing guards, protecting a lead), Complex mid-game scenarios, and Critical high-stakes situations.

Clarity and Viewpoint: All selected images were high-resolution and taken from a consistent, behind-the-hacker viewpoint, simulating the visual perspective of the athlete in the wheelchair.

Variability in Complexity: The set included scenarios with varying levels of complexity, defined by the number of stones in play (ranging from 3 to 10 stones) and the strategic difficulty of the required shot.

These rigorous criteria ensured that the selected stimuli were representative of the dynamic visual and cognitive challenges faced by elite wheelchair curling athletes during actual competition.

Example Figure 1 (Defense)

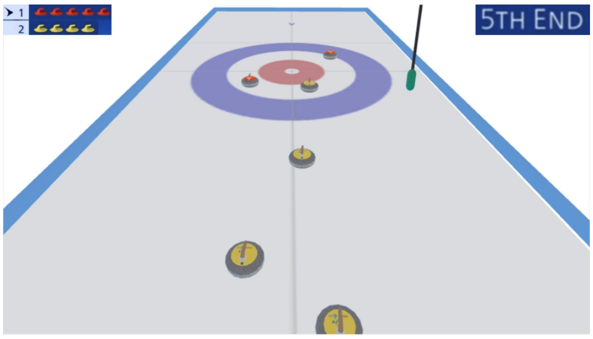

Example Figure 1 (Defense): A typical mid-game defensive scenario in the 5th end. With few stones in play and the score likely close, the red stone (our team) is positioned to control the center, reflecting a "containment" strategy that limits the opponent's scoring opportunities.

Example Figure 2(Defense)

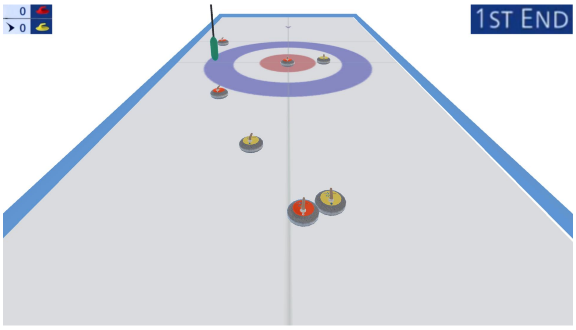

Example Figure 2 (Defense): An early-game defensive setup in the 1st end. The placement of a center guard is a conservative tactic aimed at controlling the game's pace and waiting for an opponent's error, which is the core of the "containment" philosophy.

Example Figure 3(Offense):

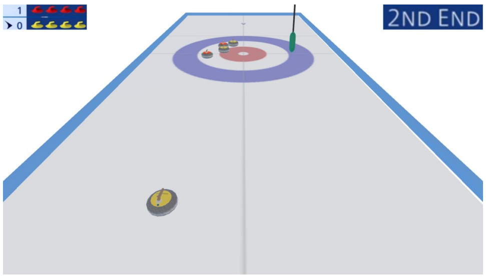

Example Figure 3 (Offense): A developing offensive opportunity in the 2nd end. After a potential opponent error, the house is open for our red team to score. This scenario tests the athlete's ability to switch from containment to attack and capitalize on the opportunity.

Example Figure 4(Offense):

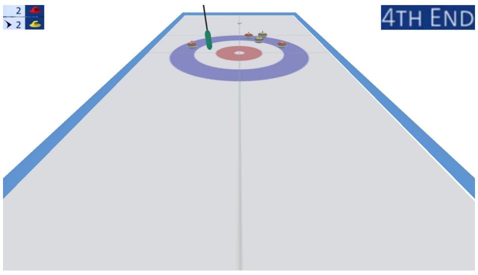

Example Figure 4 (Offense): A clear offensive situation in the 4th end. The opponent's stones are not in ideal defensive positions, providing a direct path for our red team to execute a scoring shot.
